# Awareness About Fall Risk and Measures of Fall Prevention Among Older Adults in Buraidah, Saudi Arabia

**DOI:** 10.7759/cureus.63328

**Published:** 2024-06-27

**Authors:** Raghad A Almesned, Saulat Jahan

**Affiliations:** 1 Family Medicine Academy, Qassim Health Cluster, Buraidah, SAU

**Keywords:** saudi arabia, prevention, awareness, elderly, falls

## Abstract

Background

Falls, particularly common among the elderly, pose significant health risks and mortality rates worldwide. Factors such as decline related to old age, gender, medical conditions, and environmental hazards contribute to falls. Prevention strategies focus on environmental modifications, exercise programs, medication reviews, and vitamin D supplementation to reduce fall risks and improve outcomes. This study aims to investigate the awareness of fall risk and measures of fall prevention among older adults in Buraidah, Qassim, Saudi Arabia, and examines the relationship between the level of awareness and various sociodemographic factors.

Methodology

This cross-sectional study was conducted among elderly patients at primary healthcare centers in Buraidah, Qassim province, Saudi Arabia. Data were collected via an interviewer-administered questionnaire assessing awareness and prevention of falls. Data were cleaned in Excel (Microsoft Corp., Redmond, WA, USA) and analyzed using SPSS version 29 (IBM Corp., Armonk, NY, USA). A linear regression model was used to determine the association. Statistical significance was established at a p-value of 0.05 or lower.

Results

Our study included 280 elderly participants, of whom 58.2% were female. The mean age was 63.7 years (SD = 4.9), and 34.6% had a bachelor’s degree. Regarding fall awareness, 81.4% acknowledged preventability. Notable preventive measures included medication reviews (64.6%), eye examinations (85.7%), physical activity (82.2%), vitamin D supplementation (76.8%), and home safety devices (97.5%). Regarding fall prevention, 61.8% underwent medical examinations annually, and 65.4% had vision checkups. Higher awareness about fall risks was associated with female gender (β = 1.394, 95% confidence interval (CI) = 0.199 to 2.589, p = 0.022), higher education (β = 0.931, 95% CI = 0.549 to 1.314, p < 0.001), and chronic diseases (β = -1.935, 95% CI = -3.313 to -0.556, p = 0.006).

Conclusions

Our study demonstrates significant awareness among elderly participants regarding fall preventability and measures. Females and those with higher education levels had higher levels of awareness. These findings highlight the importance of targeted interventions to increase awareness and preventive measures among elderly populations.

## Introduction

Fall is described as an event when a person unintentionally comes to rest on the ground or a lower level without any evident underlying external cause, for example, stroke or sudden loss of balance. It is a type of unintentional injury that is common among older individuals. Falls can lead to fatal and non-fatal outcomes [1].

According to the World Health Organization (WHO), falls are a common health problem and can be considered the second leading cause of deaths occurring due to unintentional injuries. Data from the WHO estimated that more than 680,000 deaths are caused by fall-related injuries, with the majority (more than 80%) of these deaths reportedly occurring in the developing regions of the world [2].

Although falls can lead to lethal outcomes in any age group, they affect the elderly more. A higher frequency of fall-related injuries is reported in children, athletes, and elderly persons. People over the age of 60 years have higher rates of deaths occurring due to fall-related injuries. Increasing age may lead to declining functionalities in the body, especially the coordination among different organs becoming weaker. For instance, vision, hearing abilities, muscular strength, control, and body balance maintenance are required to regulate the normal gait of the body. A decline in these functions with increasing age along with comorbid medical issues and illnesses accompanied by the side effects of medications may increase the risk of falls in the elderly [1-3].

Management strategies for falls primarily focus on preventing incidents among the population at risk. These strategies are designed to intervene in the environmental factors to ensure safe and fall-resistant surroundings around the elderly. Home environments are analyzed by professionals to estimate the fall risk and interventions are proposed. These interventions may require basic changes such as the introduction of assistive devices, non-slipping shoes, walking aids, and safety alarms. More complex interventions may involve improving the living place to enhance movement and flexibility while reducing the hazardous factors that may lead to serious outcomes of a fall-related incident [1,2,4]. Further, exercise programs that involve training for balance maintenance, functional exercises, and resistance exercises may lead to a reduced frequency of fall incidents [5].

Medication review is an important intervention to prevent falls in high-risk individuals. Patients with a history of falls should be prescribed carefully, especially when elderly patients have to use antidepressants, sedatives, or psychotropic drugs as they have been linked to increased risk of falls. Moreover, vitamin D supplementation can be regarded as an intervention when patients at risk of falls are deficient in vitamin D [1,2].

Falls can lead to unwanted health-related consequences with mild-to-severe outcomes in any age group, particularly in the elderly. Therefore, evaluation of fall risk is an important factor when providing care to an elderly individual in healthcare facilities as well as at home. For this purpose, it is important to understand the awareness level of the elderly population about fall risk and their perception of themselves as being at risk of falls. Therefore, this study was designed to identify the areas where the elderly lack knowledge about the risk of falls. The results of this study will help in understanding the attitudes and behaviors of the elderly that increase their risk of falls, leading to guidance in the development of appropriate interventional strategies.

The objectives of the study were to explore the awareness of fall risk and measures of fall prevention among older adults in Buraidah, Qassim, Saudi Arabia, and to explore the relationship between the level of awareness and different sociodemographic factors.

## Materials and methods

Study design and setting

A cross-sectional study was conducted among the elderly population attending primary healthcare centers (PHCCs) in Buraidah, Qassim, Saudi Arabia. Patients aged 60 years and above were included in the study while those in wheelchairs or handicapped were excluded. Furthermore, elderly patients with cognitive impairment (not able to answer questionnaires) were also excluded from the study.

Sample size and sampling technique

The sample size was calculated using OpenEpi statistical software. The reference criteria of perceived susceptibility to falls were taken from a previous study [6], which showed awareness regarding falls among 75.5% of the study participants. Based on a 95% confidence interval (CI) and a 5% margin of error, the calculated sample size was 284 individuals. The largest five PHCCs in Buraidah City were selected for the study. All elderly patients attending the selected PHCCs were invited to participate according to participant selection criteria.

Data collection tools

The study was conducted using an interviewer-administered questionnaire which was distributed after excluding patients who did not meet the inclusion criteria. The aim of the study was clearly explained to the study participants. The principal investigator collected the data by interviewing the participants. A structured Arabic language questionnaire was used. The questionnaire was adapted from previous studies [7-10]. It was an interviewer-administered, semi-structured questionnaire. The questionnaire included questions regarding sociodemographic characteristics of the participants such as age group, sex, nationality, and residence. Moreover, questions regarding awareness of fall risk and measures of fall prevention among older adults were also included. There were eight questions for assessing awareness while 10 questions for assessing the preventive measures adopted by the study participants. The five-point Likert scale was used for the statements regarding awareness while the response options for the adoption of preventive measures were “Yes” or “No.” The questionnaire was pretested in a pilot study of 20 participants whose data were not included in the study. Based on respondents’ comments, modifications were made to the questionnaire to ensure clarity and easy understanding of the questions.

Data analysis

The data were collected through Google Forms and transferred to Microsoft Excel (Microsoft Corp., Redmond, WA, USA). After coding in Microsoft Excel, the data were transferred to SPSS version 29 (IBM Corp., Armonk, NY, USA) for analysis. A comprehensive statistical analysis was conducted on the dataset, encompassing both descriptive and inferential methodologies. First, a descriptive analysis was conducted to summarize the demographic characteristics of the participants, which included age, gender, and other features. This provided an overview of the study population. Subsequently, inferential analyses (linear regression model) were used to identify the associations with awareness and prevention levels. Statistical significance was established at a p-value of 0.05 or lower.

Ethical considerations

Ethical approval for the study was obtained from the Qassim Regional Research Ethics Committee (approval number: 607/45/3772). Informed consent was obtained from the study participants. All data were kept confidential and used only for research purposes.

## Results

Our study included 280 elderly participants as we could not reach the calculated sample size of 284 because of time constraints. Regarding gender distribution, 41.8% (n = 117) were male and 58.2% (n = 163) were female. The mean age was 63.7 years (SD = 4.9), ranging from 60 to 98 years. Education levels varied, with 11.1% being illiterate, 12.5% having primary education, and 34.6% holding a bachelor’s degree. The majority were married (85.4%, n = 239), while 79.2% (n = 221) reported having chronic diseases. A significant proportion (73.2%, n = 205) used regular medications, with 30.4% (n = 85) taking one to two medications. Regarding occupation, 57.5% (n = 161) of participants were retired and 30% (n = 84) were housewives. Moreover, 85.0% (n = 238) lived in their own house with family. The prevalence of smoking was low (7.1%, n = 20), with 4.3% (n = 12) being former smokers (Table 1).

**Table 1:** Sociodemographic characteristics of the study participants (n = 280).

Sociodemographic characteristics		Frequency (n = 280)	Percent (%)
Gender	Male	117	41.8
	Female	163	58.2
Age	Mean (SD)	63.7 (4.9)	
	Range	60-98	
Education level	Illiterate	31	11.1
	Primary education	35	12.5
	Intermediate education	19	6.8
	Secondary education	62	22.1
	Bachelor	97	34.6
	Master/Doctoral	36	12.9
Marital status	Divorced	5	1.8
	Married	239	85.4
	Widowed	36	12.9
Having chronic disease	No	58	20.8
	Yes	221	79.2
Use regular medications	No	75	26.8
	Yes	205	73.2
Number of regular medications	None	75	26.8
	1-2	85	30.4
	3-4	57	20.4
	More than 4	63	22.5
Occupational status	Employed	23	8.2
	Private business	12	4.3
	Retired	161	57.5
	Housewife	84	30.0
Status of living	Lives alone in their own house	13	4.6
	Lives alone in their rented house	2	0.7
	Lives in their own house with family members	238	85.0
	Lives in their rented house with family members	27	9.6
Smoking status	No	248	88.6
	Yes	20	7.1
	Ex-smoker	12	4.3

**Figure 1:**
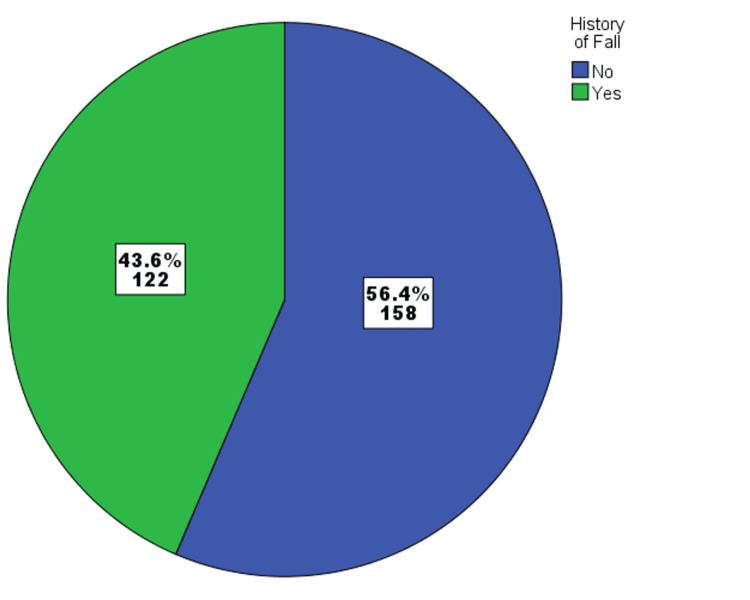
History of falls among study participants (n = 280).

Figure 1 shows the history of falls among the study participants. Among them, 56.4% (n = 158) reported no history of falls for the last two years, while 43.6% (n = 122) reported experiencing at least one fall in the last two years.

Table 2 shows the awareness levels regarding the risk of falls among the study participants. A high proportion, 81.4% (n = 228), agreed that falls in older adults can be prevented, with 35.7% strongly agreeing and 45.7% agreeing. Similarly, the participants agreed to the concern about falls among older adults, with 87.8% (n = 246) acknowledging it, 49.6% strongly agreeing, and 38.2% agreeing. Concerning medications, 66.1% (n = 185) were either neutral or disagreed on whether taking ≥4 medications daily increased the risk of falling. Furthermore, a substantial proportion of the respondents were aware of the importance of annual medication reviews, annual eye examinations, and being active for at least two and a half hours a week in reducing fall risk. The potential of daily vitamin D supplementation and the effectiveness of installing and using home safety devices in fall prevention was also recognized by the majority of the study participants.

**Table 2:** Awareness of the risk of falls among geriatric patients attending primary healthcare centers in the Qassim region (n = 280).

Statement		Frequency (n = 280)	Percent (%)
Falls in older adults can be prevented	Strongly disagree	1	0.4
	Disagree	14	5.0
	Neutral	37	13.2
	Agree	128	45.7
	Strongly agree	100	35.7
Falling is a concern in older adults	Strongly disagree	0	0.0
	Disagree	20	7.1
	Neutral	14	5.0
	Agree	107	38.2
	Strongly agree	139	49.6
Taking ≥4 medications daily increases the risk of falling	Strongly disagree	4	1.4
	Disagree	49	17.5
	Neutral	136	48.6
	Agree	70	25.0
	Strongly agree	21	7.5
Annual review of medications with health professionals reduces the risk of falling	Strongly disagree	2	0.7
	Disagree	24	8.6
	Neutral	73	26.1
	Agree	123	43.9
	Strongly agree	58	20.7
Annual eye examination reduces the risk of falling	Strongly disagree	1	0.4
	Disagree	17	6.1
	Neutral	22	7.9
	Agree	147	52.5
	Strongly agree	93	33.2
Being active for at least two and a half hours a week reduces the risk of falling	Strongly disagree	2	0.7
	Disagree	10	3.6
	Neutral	38	13.6
	Agree	127	45.4
	Strongly agree	103	36.8
Daily vitamin D supplementation reduces the risk of falling	Strongly disagree	0	0.0
	Disagree	7	2.5
	Neutral	58	20.7
	Agree	141	50.4
	Strongly agree	74	26.4
Installing and using home safety devices reduces the risk of falling	Strongly disagree	0	0.0
	Disagree	2	0.7
	Neutral	5	1.8
	Agree	89	31.8
	Strongly agree	184	65.7

**Figure 2:**
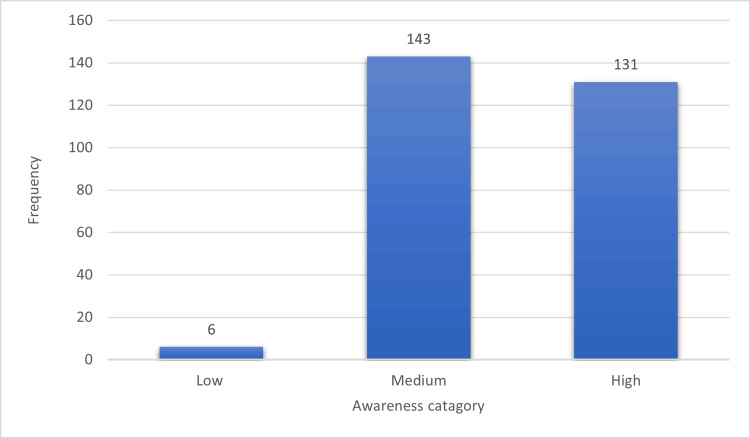
Study participants’ levels of awareness about the risk of falls (n = 280). Level of awareness: total possible score = 40. Low = 24 or less; medium = 25-32; high = 33-40.

Figure 2 shows the distribution of different categories of awareness about the risk of falls among the 280 participants. Out of the total 40, six (2.1%) individuals scored 24 or less and were categorized as having low awareness, while 143 (51.1%) individuals had medium awareness scoring 25-32, and 131 (46.8%) individuals were classified as having high awareness by achieving scores ranging 33-40.

Table 3 shows preventive measures adopted by the study participants. A substantial proportion of patients (61.8%, n = 173) had annual medical examinations. Similarly, 65.4% (n = 183) of patients followed annual vision examinations. Regarding medication management, 32.5% (n = 91) reviewed medication side effects with healthcare providers if taking ≥4 medications. Concerning physical activity, 35.0% (n = 98) engaged in regular exercise. Moreover, 56.1% (n = 157) ensured adequate intake of vitamin D and calcium and 43.2% (n = 121) had poorly lit areas in the living house. Regarding home safety, the majority had stairs with railings (84.3%, n = 236), but fewer had grab bars or rails installed in baths/showers (27.5%, n = 77), rubber bathmats (34.6%, n = 97), or raised toilet seats (74.6%, n = 209).

**Table 3:** Prevention of falls among geriatric patients attending primary healthcare centers in the Qassim region (n = 280).

Preventive measures for fall prevention		Frequency (n)	Percent (%)
Have an annual medical examination	No	107	38.2
	Yes	173	61.8
Have an annual vision examination	No	97	34.6
	Yes	183	65.4
If taking ≥4 medications, review of medication side effects with the healthcare provider	No	55	19.6
	Yes	91	32.5
	Not applicable	134	47.9
Have regular physical exercise up to 30 minutes of moderate-intensity activity on five days of the week	No	182	65.0
	Yes	98	35.0
Eat well and include adequate vitamin D and calcium intake	No	123	43.9
	Yes	157	56.1
Have any poorly lit areas in your living place	No	159	56.8
	Yes	121	43.2
Have a stair with railings on one or both sides	No	44	15.7
	Yes	236	84.3
Have grab bars or rails installed in the bath/shower	No	203	72.5
	Yes	77	27.5
Have a rubber bathmat or a non-slip surface on the bath/shower floor	No	183	65.4
	Yes	97	34.6
Have a raised toilet seat installed	No	71	25.4
	Yes	209	74.6

**Figure 3:**
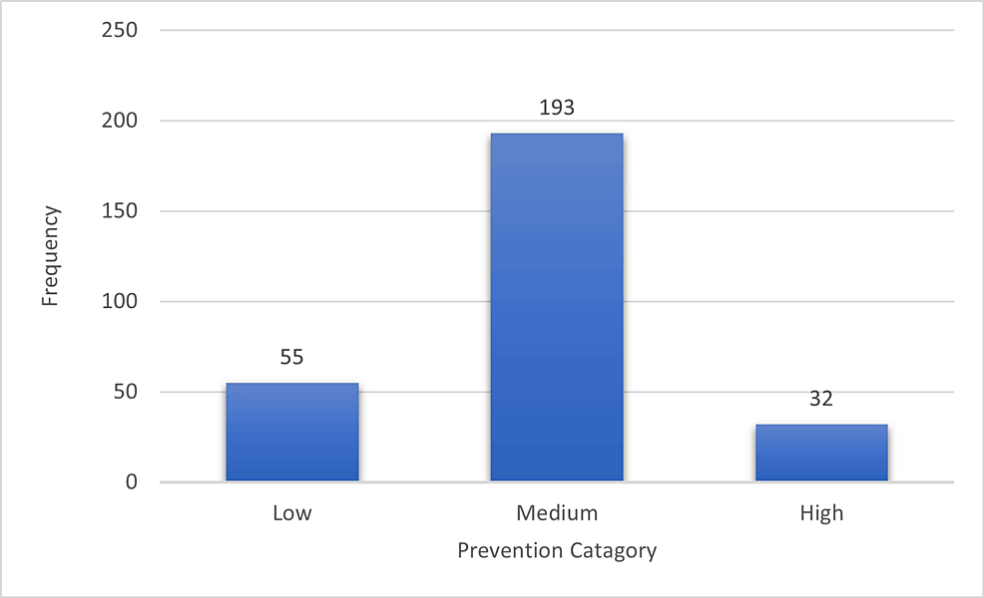
Different levels of prevention from falls among study participants (n = 280). Level of prevention: Total possible score = 10. Low = 3 or less; medium = 4-7; high = 8-10.

Figure 3 shows the different levels of fall prevention among geriatric patients. A total of 55 (19.6%) individuals were categorized as adopting low-level preventive measures with a score of 3 or less out of the total 10, while the majority (68.9%) had a medium level of prevention with scores ranging 4-7. Only 32 (11.4%) individuals were classified as having a high level of preventive measures (8-10).

When asked whether the study participants received guidance about fall risk and its prevention, 211 (75.4%) respondents reported not receiving such guidance. Regarding sources of information about fall risk and its prevention, the majority, 184 (65.7%), reported not having any source of information specifically related to fall risk and prevention. Family, friends, or social media served as the source of information for 74 (26.4%) individuals. Healthcare workers were cited as the source by 15 (5.3%) individuals, while 7 (2.5%) individuals mentioned other sources.

Gender (β = 0.175, p = 0.022), higher education (β = 0.367, p < 0.001), and chronic diseases (β = -0.200, p = 0.006) were significantly associated with the awareness regarding the risk of falls. Females and those with higher education had better awareness about fall risk, while those having chronic diseases had low awareness levels. Age (β = 0.021, 95% CI = -0.084 to 0.125, p = 0.696), body mass index (BMI) (β = 0.071, 95% CI = -0.032 to 0.173, p = 0.177), marital status (β = 0.008, 95% CI = -1.288 to 1.303, p = 0.991), medication use (β = -0.190, 95% CI = -1.787 to 1.408, p = 0.815), occupation (β = 0.056, 95% CI = -0.623 to 0.735, p = 0.871), living arrangement (β = 0.078, 95% CI = -0.752 to 0.909, p = 0.853), history of falls (β = -0.401, 95% CI = -1.296 to 0.494, p = 0.379), and smoking status (β = -0.459, 95% CI = -1.485 to 0.568, p = 0.380) did not have significant association with awareness regarding the risk of falls. None of the sociodemographic factors were significantly associated with the adoption of preventive measures against falls: age (β = 0.014, 95% CI = -0.042 to 0.071, p = 0.616), gender (β = 0.176, 95% CI = -0.467 to 0.818, p = 0.591), educational level (β = 0.131, 95% CI = -0.075 to 0.336, p = 0.212), BMI (β = -0.024, 95% CI = -0.079 to 0.032, p = 0.401), marital status (β = 0.047, 95% CI = -0.649 to 0.743, p = 0.894), medication use (β = 0.469, 95% CI = -0.389 to 1.327, p = 0.283), occupation (β = -0.084, 95% CI = -0.449 to 0.281, p = 0.652), living arrangement (β = -0.151, 95% CI = -0.597 to 0.296, p = 0.506), history of falls (β = -0.137, 95% CI = -0.618 to 0.344, p = 0.575), and smoking status (β = -0.086, 95% CI = -0.637 to 0.466, p = 0.760).

## Discussion

Falls, which are common among older individuals, are a major global cause of mortality and unintentional injury [10]. Age-related declines in coordination and other functions increase fall risk, with factors such as gender, medical conditions, and medication also playing important roles [11]. Prevention strategies include environmental modifications, exercise programs, medication reviews, and vitamin D supplementation [12]. Thus, our study aimed to assess the awareness of fall risk and measures of fall prevention among older adults in Buraidah, Qassim, Saudi Arabia. Our results provide valuable insights into the demographic characteristics, history of falls, awareness levels, preventive measures, and sources of information among the study participants.

Our study had a balanced representation of gender, with a slight majority of females. This gender distribution aligns with the global trend of women tending to live longer than men [13] and being more prone to age-related conditions, including falls [14]. Moreover, the mean age of the participants was 63.7 years and above, with a significant proportion having a bachelor’s degree, reflecting the educational landscape of Saudi Arabia. Chronic diseases and regular medication use highlight the importance of fall prevention strategies in managing comorbidities and medication-related risks. Similarly, Denfeld et al. (2022) reported that medications, heart disease, orthostatic hypotension, and frailty increase fall risk, especially in older adults with cardiovascular issues [15].

In this study, 43.6% of participants reported a history of falls during a period of two years, highlighting the prevalence of falls among older adults in the region. Almegbel et al. (2018) found that the annual prevalence of falls among the elderly in Riyadh was 49.9% [8]. Another study conducted in Tabuk, Saudi Arabia, reported the fall prevalence as 25.3% during a one-year period [16]. Falls can lead to serious injuries, reduced mobility, and increased healthcare utilization, emphasizing the need for effective prevention measures.

Our study assessed awareness levels regarding fall risk and prevention among geriatric patients. Participants showed positive attitudes toward fall prevention, with widespread recognition of its seriousness. Many believed in the effectiveness of preventive measures such as medication reviews, eye examinations, physical activity, and vitamin D supplementation, aligning with previous research [15]. Henriksen et al. (2008) suggested various other standard fall prevention interventions such as remaining with the patient while toileting, observing/rounding every hour, and reorienting confused patients. Some other fall prevention interventions suggested in the literature include remaining with the patient while toileting and observing/rounding every hour, and notifying receiving areas of high fall risk [17].

This study also evaluated various preventive measures adopted by geriatric patients in the region. It is an encouraging finding that a substantial proportion of participants underwent annual medical and vision examinations. A vision examination is crucial for the elderly as eye problems such as glaucoma, retinal degeneration, and best-corrected visual acuity of the better eye are reported as independent risk factors for falls in elderly patients with visual Impairment [18]. Only a third of participants reviewed medication side effects with healthcare providers if taking multiple medications, indicating a need for improved medication safety practices among older adults to prevent falls. Moreover, the result of linear regressions in our study showed that medication use was not significantly associated with fall prevention behaviors or fall awareness. In contrast, a study by Ming et al. (2021) showed medication review’s efficacy, alone or combined, in preventing fall-related injuries among older adults [19]. In this study, the proportion of participants engaging in regular physical exercise was relatively low, suggesting potential opportunities for promoting physical activity as a fall prevention strategy. Home safety measures were moderately adopted, with the majority having stairs with railings but fewer implementing grab bars or rails in baths/showers or rubber bathmats. Similarly, Campani et al. (2021) stated that assessing and modifying home arrangements is an effective preventive measure against falls and fall‐related injuries [20]. These findings emphasize the importance of addressing environmental hazards and promoting home safety modifications to prevent falls among older adults.

Our study reveals that a substantial proportion (75.4%) of geriatric patients did not receive guidance about fall risk and its prevention, with family, friends, and social media being key sources of information. Healthcare workers play a minor role in providing fall prevention guidance, highlighting the need for increased awareness and improved communication between healthcare providers and patients.

This study also showed a significant association of higher awareness about fall risks with gender, higher education, and chronic diseases. Females and respondents with higher education levels had greater awareness, while the presence of chronic diseases was associated with lower awareness levels. In agreement with our study, Patton et al. (2022) also found that older adult females had more awareness about fall risks [21].

Our study focused on preventive measures taken by the elderly population. In a literature search, we were not able to find such a study conducted in Saudi Arabia. However, our study has several limitations. Due to resource constraints and limited time available, we had to stop data collection very close to achieving the calculated sample size. We were short by just four participants, which is not expected to significantly affect the results of the study. The self-reported data may introduce recall bias and social desirability bias. The cause-effect relationship cannot be established because of the study’s cross-sectional design. The study was conducted in one city of a province limiting its generalizability to other populations.

## Conclusions

Our study provides insights into the awareness of fall risk and the adoption of measures of fall prevention among older adults in Buraidah, Qassim, Saudi Arabia. The findings highlight the importance of promoting awareness and designing evidence-based intervention strategies to reduce the burden of falls among older adults. Healthcare providers may receive training in communication skills to play their role in educating patients about fall risk and prevention strategies.
